# Level of adherence to option B+ program and associated factors among HIV-positive women in Ethiopia: A systematic review and meta-analysis

**DOI:** 10.1371/journal.pone.0298119

**Published:** 2024-04-25

**Authors:** Alemu Degu Ayele, Bekalu Getnet Kassa, Gedefaye Nibret Mihretie, Habtamu Gebrehana Belay, Dagne Addisu Sewyew, Abenezer Melkie Semahegn, Enyew Dagnew Yehuala, Gebrehiwot Ayalew Tiruneh, Lebeza Alemu Tenaw, Abrham Debeb Sendekie, Adanech Getie Teffera, Eden Workneh Aychew, Yismaw Yimam Belachew, Tewachew Muche Liyeh, Mulugeta Dile Worke

**Affiliations:** 1 Department of Midwifery, College of Health Sciences, Debre Tabor University, Debre Tabor, Ethiopia; 2 School of Public Health, College of Health Sciences, Woldia University, Woldia, Ethiopia; 3 College of Medicine and Health Sciences, Wachemo University, Hossana, Ethiopia; 4 School of Medicine, College of Health Sciences, Debre Tabor University, Debre Tabor, Ethiopia; Auckland University of Technology, NEW ZEALAND

## Abstract

**Background:**

Despite policy initiatives and strategic measures highly focused on preventing mother-to-child transmission through the implementation of the Option B+ program, adherence to the treatment is still challenging. The level of adherence and determinants of Option B+ program utilization reported by different studies were highly inconsistent in Ethiopia. Hence, this systematic review and meta-analysis aimed to estimate the pooled prevalence of adherence to the Option B+ program and its predictors among HIV-positive women in Ethiopia.

**Methods:**

PubMed, Google Scholar, EMBASE, HINAR, Scopus, and Web of Sciences were searched for published articles from March 2010 to March 2022. The pooled prevalence of adherence was estimated using a weighted DerSimonian-Laird random effect model. The I^2^ statistics was used to identify the degree of heterogeneity. Publication bias was also assessed using the funnel plot and Egger’s regression test.

**Results:**

A total of 15 studies were included. The pooled estimate of the option B+ program among HIV-positive women in Ethiopia was 81.58% (95% CI: 77.33–85.84). Getting social and financial support (AOR = 3.73, 95% CI: 2.12, 6.58), disclosure of HIV status to partners (AOR = 2.05, 95% CI: 1.75, 2.41), time to reach a health facility (AOR = 0.33, 95% CI: 0.16, 0.67), receiving counseling on drug side effects (AOR = 4.09, 95% CI: 2.74, 6.11), experience of drug side effects (AOR = 0.17, 95% CI: 0.08, 0.36), and knowledge (AOR = 4.73, 95% CI: 2.62, 8.51) were significantly associated with adherence to the Option B+ program.

**Conclusion:**

This meta-analysis showed that the level of adherence to the Option B+ program in Ethiopia is lower than the 95% level of adherence planned to be achieved in 2020. Social and financial support, disclosure of HIV status, time to reach the health facility, counseling, drug side effects, and knowledge of PMTCT were significantly associated with option B+ adherence. The findings of this meta-analysis highlight that governmental, non-governmental, and other stakeholders need to design an effective strategy to scale up the level of disclosing one’s own HIV status, access health facilities, improve knowledge of PMTCT, and counsel the potential side effects of Option B+ drugs, and advocate the program to reduce the multidimensional burden of HIV/AIDS.

**Trial registration:**

**Prospero registration:** CRD42022320947. https://www.crd.york.ac.uk/prospero/display_record.php?ID=CRD42022320947.

## Introduction

Mother-to-child transmission (MTCT) is the most significant source of human immunodeficiency virus (HIV) infection in children, which mainly occurs during pregnancy, the intrapartum period, or after delivery through breastfeeding [1]. Approximately 15–30% of infants born from HIV-positive women will become infected with HIV during pregnancy and the intrapartum period, and around 5–15% of newborns acquire the virus after delivery through breastfeeding [2,3]. Among the four prongs of comprehensive HIV/AIDS prevention programs endorsed by the World Health Organization (WHO), the prevention of mother-to-child transmission (PMTCT) platform is the third intervention intended for the well-being of women living with HIV. It reduces the risk of transmission to their infants by providing appropriate antiretroviral treatment (ART) [4–6].

Without PMTCT intervention, 50% of children born to HIV-positive women will die before their second birthday, and 80% of them have a risk of under-five mortality [2,7]. Moreover, HIV infection creates a lifelong chronic condition in newborns, including a shorter life expectancy, and contributes to substantial human, social, and economic costs [2].

In 2013, the WHO consolidated guidelines recommended that every pregnant and breastfeeding woman infected with HIV should initiate antiretroviral treatment. However, these guidelines still offered the option to either continue antiretroviral treatment for lifelong therapy or to stop the treatment after delivery when the risk of mother-to-child transmission becomes low [8]. Hence, a revised recommendation in 2015 advocates the option B+ program, which means that all pregnant and breastfeeding HIV-infected women should initiate ART as soon as possible and remain on lifelong treatment regardless of gestational age, their CD4 cell count, and the clinical stage of the disease to minimize the risk of mother-to-child transmission of HIV AIDS [9]. Option B+ program follows the principles of the test and treat approach, meaning that initiating ART drugs on the same day of HIV diagnosis has a significant benefit in settings where there is a high rate of HIV prevalence, fertility, and a long duration of breastfeeding [9].

Afterwards, many countries adopted the Option B+ program and included it in the global plan toward the elimination of new HIV infections among children by 2015 and keeping their mothers alive [10]. This global plan spurred remarkable progress and reduced the rate of new HIV infections among children by 60% in the highest-burden Sub-Saharan African countries. However, approximately 110, 000 children and 150, 000 children were newly infected with HIV in Sub-Saharan Africa and worldwide in the same year, 2015, respectively [11].

Ethiopia has been implementing the Option B+ program since 2013 and has advocated the significance of early initiation of ART to reduce vertical virus transmission. A combination of three ARV drugs (TDF+3TC+EFV) is the preferred dosage regimen for pregnant and lactating women [12]. In agreement with the Sustainable Development Goals, the global community set an ambitious target of ending the AIDS epidemic by 2030 [13]. To achieve this goal, the number of new HIV infections and AIDS-related deaths will need to decline by 90% compared to 2010 [14].

A new super-fast track has been established to end the AIDS-related pandemic among children, adolescents, and young women by 2020. This super-fast-track approach plans to reduce the number of newly infected children to less than 40, 000 by 2018 and 20, 000 by 2020 to reach and sustain 95% of pregnant women living with HIV with lifelong HIV treatment by 2018 [15].

Treatment adherence is an essential parameter for staying healthy and eliminating new infections. Option B+ is the widely advocated, emphasized, influential, and best strategy for staying healthy for an extended period and removing new infections. However, women’s adherence to the program is still challenging [16,17]. The success of PMTCT depends on the proportion of women who adhere to treatment and are retained in care, but there was a significantly high attrition rate due to loss to follow-up (LTFU), followed by mortality [18–21].

Despite the fact that the government of Ethiopia and other stakeholders have been designing and implementing different strategies and activities, including the Option B+ program, safe delivery service, and exclusive breast feeding, to minimize the rate of MTCT, the 2020 target plan to maximize the level of drug adherence to 95% has not yet been achieved [22].

Various primary studies regarding the Option B+ program have been conducted in different regions of Ethiopia. These detached studies showed that the level of option B+ program adherence among HIV-positive women in the country ranges from 60% in Oromia [23] to 95.1% in Tigray [24]. The report of these detached studies showed that there is a great variation and inconsistency related to adherence to the program among HIV-positive women throughout the country. The reasons for the above variation and inconsistency have not yet been investigated. There is also one systematic review done in East Africa using 14 primary articles, of which only eight were from Ethiopia, which might be insufficient to provide generalized information for the Ethiopian context [25]. Still, the current review was conducted entirely in Ethiopia, using a total of 15 primary studies. In addition, the former review only identified four factors, whereas the current review introduces extra factors that were significantly associated with adherence to the Option B+ program, including time to reach a health facility, experience with drug side effects, and knowledge of PMTCT, which are crucial for stakeholders working in the area to improve the overall implementation of the program. Hence, this study aimed to estimate the pooled prevalence of the level of adherence to the Option B+ program and its associated factors among HIV-positive women in Ethiopia using a systematic review and meta-analysis approach.

## Material and methods

### Study design and protocol

This study used a systemic review and meta-analysis approach of relevant articles on the prevalence of option B+ program adherence and its determinants among HIV-positive women in Ethiopia. This systematic review and meta-analysis have been registered on the International Prospective Register of Systematic Reviews (PROSPERO, number CRD42022320947) and conducted according to the Preferred Reporting Items for Systematic Reviews and Meta-Analyses (PRISMA) checklist [26] (S1 Table).

### Eligibility criteria

#### Inclusion criteria

We included studies that recruited pregnant and lactating women residing in Ethiopia. Participants comprised women from diverse socioeconomic backgrounds, all ethnic groups, and those who spoke different dialects. This study included all published cross-sectional studies on the level of adherence to the Option B+ program and associated factors affecting adherence among HIV-positive women in Ethiopia. This review included studies conducted from March 2010 until March 23, 2022, and was written in English. The inclusion criteria were also guided by the PECO (population, exposure, comparison, and outcome) framework [27].

**Population:** Pregnant and lactating HIV-positive women.

**Exposure:** The exposure group consisted of all women who received the Option B+ intervention.

**Outcome:** Women’s adherence to the Option B+ program was the primary outcome of interest for this review. The second objective of this review was to identify factors that were significantly associated with the level of adherence to the Option B+ program.

#### Exclusion criteria

Studies were excluded: (1) if the study was not published in English and we were unable to access a copy translated into the English language; (2) qualitative studies; (3) case studies, as most studies lacked robust quality data to include in the analysis; (4) secondary works (e.g., review articles, commentaries, editorials, or dissertations/theses, conference abstracts that had not yet been published).

### Search strategy and data source

A comprehensive literature search was implemented using PubMed, Google Scholar, EMBASE, HINAR, Scopus, and Web of Sciences to identify relevant published studies from February 23, 2022, to March 23, 2022. We also carried out hand searches for cross-references to distinguish pertinent additional articles. Besides this, studies were also searched from all included study reference lists to find additional studies not included in our search strategies. Five authors (ADA, BGK, GNM, DAS, and HGB) searched studies exhaustively from databases using comprehensive search strategies. Initially, published studies were searched by examining the full titles (“Level of adherence to option B + program for prevention of mother-to-child transmission of HIV and associated factors among HIV positive women in Ethiopia”) and then keywords (“Level”, “adherence”, “associated factors”, “predictors”, “determinants”, “HIV positive”, “pregnant women”, “lactating women”, “women”, and “Ethiopia”). These keywords were used independently and in combination using the boolean operators “OR” or “AND.” (S2 Table).

### Identification and study selection

All identified primary studies were exported to the Endnote X7 reference manager software, and duplicated articles were excluded. In the initial screening phase, four authors (AD, BG, GN, and HG) assessed the studies independently for eligibility by reading the title, abstract, and full review using the search strategy. In the next phase of screening, those potentially eligible articles underwent full-text review to determine whether they satisfied the predetermined inclusion criteria and were assessed for duplicate records. If duplicate records were encountered, only the full-text article published was booked. Any disagreements or differences between the authors were resolved through discussion and consensus based on established criteria. If consensus could not be reached, the next six authors solved the disagreement (LAT, YYB, ADS, EWA, TML, and MDW).

#### Quality assessment

The scientific strength and quality of each enrolled original cross-sectional study were evaluated by using the Newcastle-Ottawa Scale quality assessment tool adapted for cross-sectional study quality assessments [28]. The tool has three core components; the principal component of the tool is graded from five stars and mainly highlights the methodological quality of each primary article. The second component of the tool is graded from two stars and is mainly concerned with the comparability of each study. The last component of the tool is graded from three stars and focuses on the outcomes and statistical analysis of each original study. The quality of each original study was independently assessed by four authors and weighed using these tool components. Those primary studies with a medium score (satisfying 50% quality evaluation criteria) and high quality (≥7 out of 10) were incorporated in this meta-analysis. The last four authors managed disagreements between the four authors (S3 Table).

### Data extraction

After recruiting the eligible studies, four authors (ADA, AMS, GAT, EDY, and AGT) extracted all essential data individually using a pre-tested standardized data extraction form. This form includes the primary author, year of publication, study setting, sample size, study design, response rate, the prevalence of adherence to option B+ program, significantly associated factors with adherence to option B+ program, adjusted odds ratio (AOR), and 95% CI. For the second objective (factors), the information extraction format was prepared for each specific factor, i.e., disclosure of HIV status to a partner, receiving proper counseling, time to reach the health facility, drug side effects, social and financial support, and knowledge of PMTCT. In the current analysis, variables were selected if two or more studies reported them as a significant factor. The discrepancy between the investigators in the data abstraction process was resolved by logical consensus between the four authors and finally approved by the last four authors.

### Publication bias and heterogeneity

Intensive searches (electronic/database search and manual search) were used to minimize the risk of bias. The cooperative work of the authors was also critical in reducing bias by selecting articles based on clear objectives and eligibility criteria. Visual inspection of the funnel plot graph qualitatively and Egger’s correlation test at a 5% significant level was also conducted to identify the presence of publication bias [29,30]. Moreover, point estimation and subgroup analysis were performed to investigate the random variations among the primary studies. Subgroup analysis was performed using study countries and study populations. Sensitivity analysis was also performed to identify the potential source of heterogeneity. Inverse variance (I^2^) statistics with corresponding *p-values* were used to assess heterogeneity across and within studies.

### Statistical analysis and data presentation

We used Microsoft Excel for data entry and STATA version 16 software for analysis. The random effects model based on the DerSimonian-Laird method was applied to assess variations between the studies. The results were presented using texts, tables, and forest plots with measures of effect and a 95% confidence interval. Statistical heterogeneity was tested via the I^2^ statistics at a *p*-value of ≤ 0.05 [31].

## Results

### Description of the study

**Fig 1:**
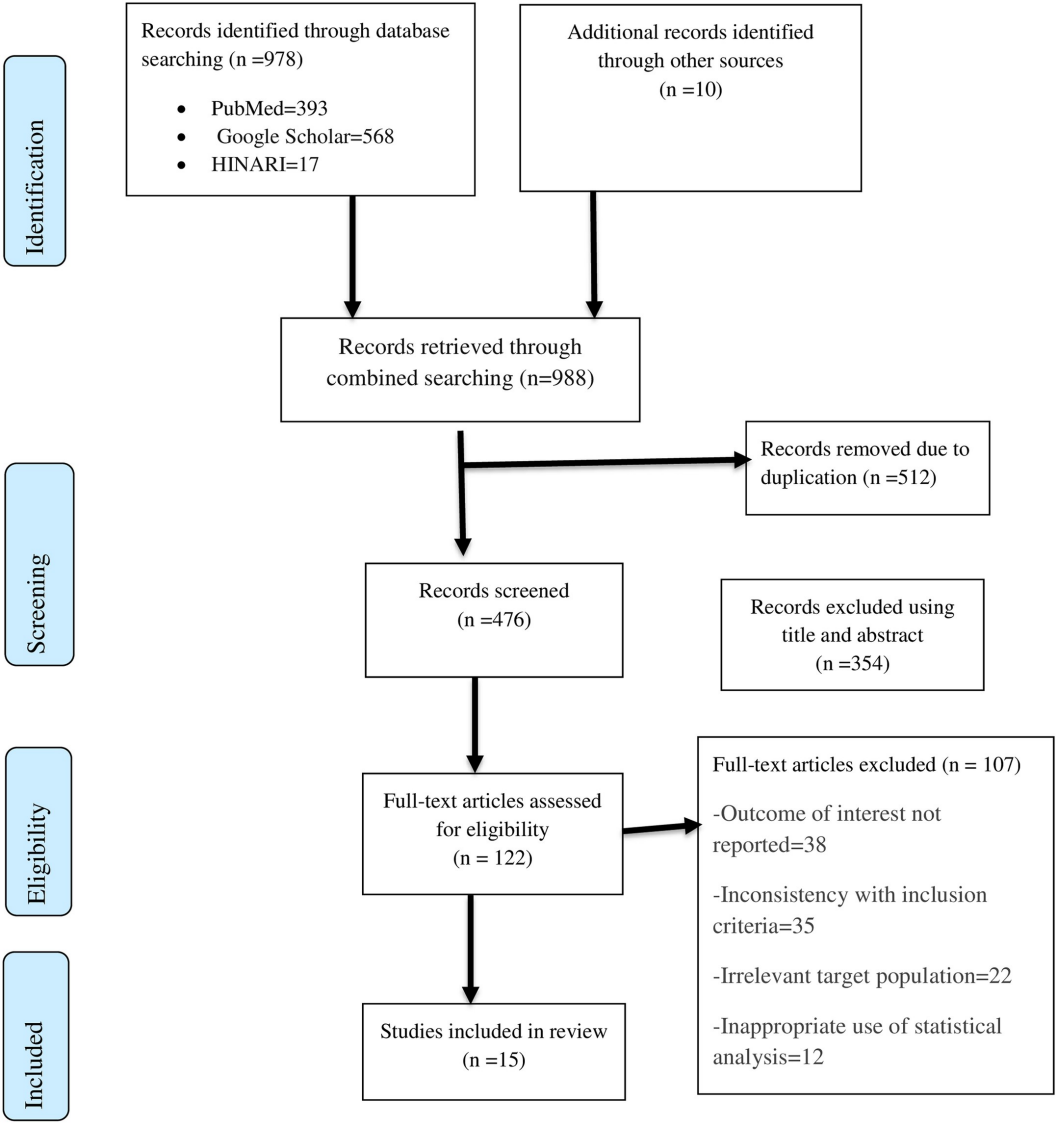
Flow chart of study selection for systematic review and meta-analysis of the level of adherence to the option B+ program among women in Ethiopia.

Among the total 988 identified studies, 512 articles were removed after reviewing their titles due to duplication, whereas the remaining 476 articles were allowed for further screening. Out of these, 354 articles were removed due to irrelevance after reviewing titles and abstracts. Then the remaining 122 articles were assessed for eligibility, and 107 of them were excluded due to the outcome of interest not being reported (38 articles), an inconsistent study report with the predetermined inclusion criteria (35 articles), an irrelevant target population (22 articles), and inappropriate use of statistical analysis (12 articles). Finally, 15 articles were included in the systematic review and meta-analysis, with a total population of 3,751 HIV-positive women (Fig 1).

### Characteristics of the included studies

All 15 eligible studies included in this systematic review and meta-analysis were cross-sectional by study design, reported in English, and performed from 2015 to 2022. The sample size ranged from 41 in the Tigray region [24] to 422 in the Oromia region [23]. Regarding the geographical distribution of the primary studies, six were from Oromia [19–21,23,32,33], four were from South Nation Nationalities and Peoples Representatives (SNNPR) [34–37], two were from Amhara [38,39], two from Tigray [24,40], and one from Harari regional states [41]. The response rate of the primary studies ranged from 94% to 100%. All fifteen included primary articles were published in reputable journals (Table 1). Lastly, the primary studies eligible for the current systematic review and meta-analysis had a quality score of 7–9 out of 10 points (S3 Table).

**Table 1:** Characteristics of included primary studies showing the level of adherence to the option B+ program and associated factors among HIV-positive women in Ethiopia.

Authors, year	Region	Study setting	Study design	Sample size	Response rate (%)	Prevalence (95%CI)	quality
Shibabaw *et al*. (2018)[24]	Tigray	institutional	C/S	41	100	95.10(94.16, 96.04)	Low risk
Asefa *et al*. (2020)[32]	Oromia	institutional	C/S	190	95	81.10(79.39, 82.81)	Low risk
Demelash *et al*. (2019)[38]	Amhara	institutional	C/S	269	100	75.00(73.11, 76.89)	Low risk
Lencha (2018)[33]	Oromia	institutional	C/S	304	96.4	82.60(80.95, 84.25)	Low risk
Fedlu *et al*. (2020) [41]	Harari	institutional	C/S	190	100	83.20(81.57, 84.83)	Low risk
Lodebo *et al*. (2017)[36]	SNNP	institutional	C/S	215	94	83.70(82.09, 85.31)	Low risk
Abdisa *et al*. (2021)[34]	SNNP	institutional	C/S	256	99.2	88.20(86.79, 89.61)	Low risk
Aferu *et al*. (2020)[35]	SNNP	institutional	C/S	103	100	66.00(63.93, 68.07)	Low risk
Tsegaye *et al*. (2016)[39]	Amhara	institutional	C/S	191	100	87.90(86.48, 89.32)	Low risk
Tesfaye *et al*. (2019)[37]	SNNP	institutional	C/S	297	97.64	81.40(79.70, 83.10)	Low risk
Ebuy *et al*.(2015)[40]	Tigray	institutional	C/S	277	95	87.10(85.64, 88.56)	Low risk
Tolossa *et al*.(2020)[19]	Oromia	institutional	C/S	330	100	90.00(88.69, 91.31)	Low risk
Wondimu *et al* (2020)[20]	Oromia	institutional	C/S	347	98.8	80.20(78.46, 81.94)	Low risk
Demissie *et al*. (2019)[21]	Oromia	institutional	C/S	319	100	81.80(80.12, 83.48)	Low risk
Degavi *et al*. (2022)[23]	Oromia	institutional	C/S	422	100	60.00(57.86, 62.14)	Low risk

C/S: Cross-sectional, SNNP: Southern Nations Nationalities Peoples.

### Level of adherence to option B+ program

**Fig 2:**
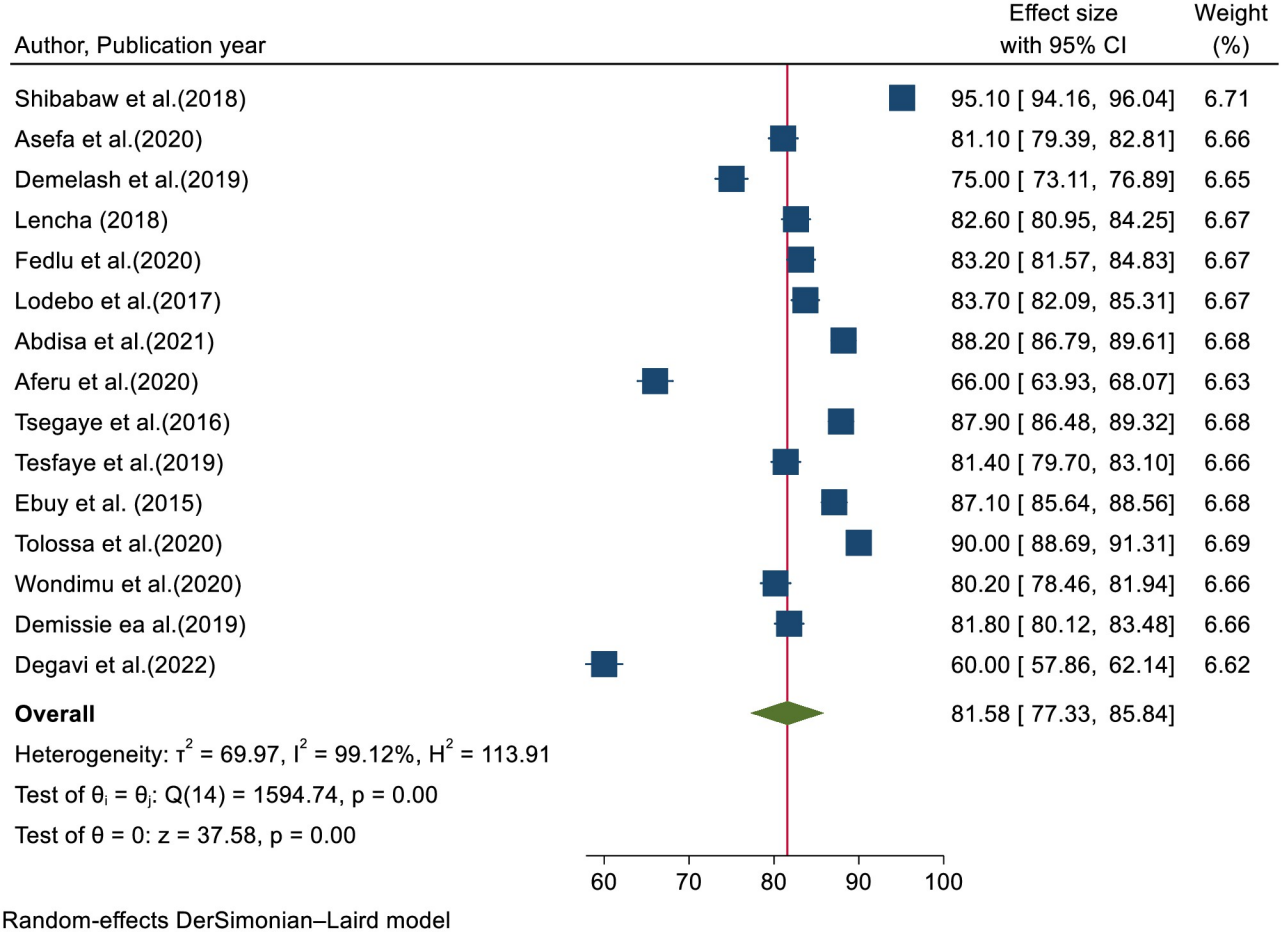
Forest plot of the pooled prevalence of level of adherence to the option B+ program among HIV-positive women in Ethiopia.

The pooled prevalence of the level of adherence to option B+ program among HIV-positive women in Ethiopia was 81.58% (95% CI: 77.33–85.84) (Fig 2).

### Heterogeneity and publication bias

There was a high level of heterogeneity across the studies, as evidenced by I^2^ statistics (I^2^ = 99.1%, P≤0.001). Hence, we used the random effect model meta-analysis approach to estimate the pooled prevalence of the level of adherence to the Option B+ program among HIV-positive women.

**Fig 3:**
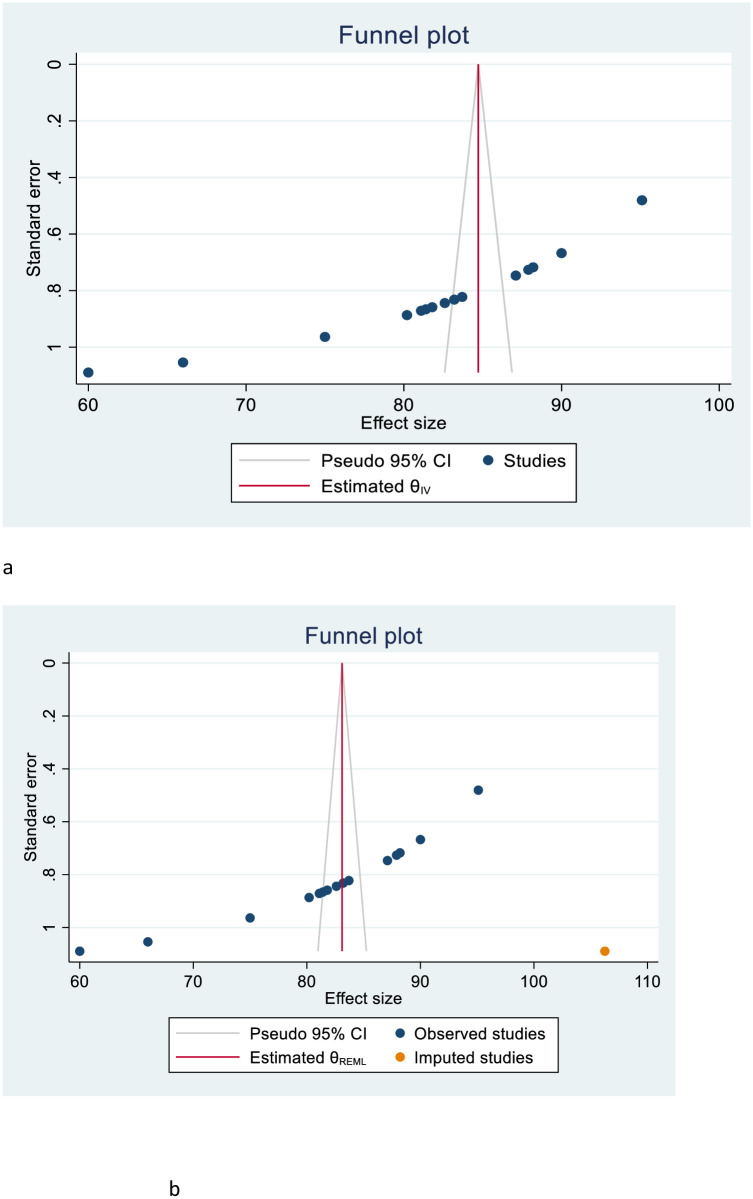
**a:** Funnel plot to test the publication bias of 15 studies. **b:** Results of the trim and fill analyses for adjusting the publication bias of the 16 studies.

Publication bias among the included primary studies was examined using both funnel plots qualitatively and Egger’s regression test quantitatively. Accordingly, the funnel plots showed an asymmetric shape, indicating publication bias (Fig 3a).

Egger’s regression test also supported the existence of publication bias across the studies (*p-value <0*.*001*). Due to this, Duval and Tweedie non-parametric trim and fill analyses were conducted to correct existing publication bias among the 15 included studies. Moreover, the publication bias was corrected when one missed study was filled in the funnel plot by trim and fill analysis. Finally, after one study was filled, a total of 16 studies were included and computed via the trim and fill analysis to produce the pooled prevalence of 79.18% (95% C: 74.02–84.34) by applying a random-effects model (Fig 3b).

### Sensitivity analysis

We carried out a leave-one-out sensitivity analysis to identify the potential source of heterogeneity observed in this study. Accordingly, the result showed that the source of heterogeneity was not reliant a particular study. The pooled prevalence of option B+ program adherence varied and ranged from 80.61% (76.73, 84.49%) to 83.12% (79.45, 86.78%) (Table 2).

**Table 2:** Sensitivity analysis of the level of option B+ program for PMTCT adherence and associated factors among HIV-positive women in Ethiopia.

Study omitted	Prevalence	95% CI
Shibabaw et al. (2018)[24]	80.61	76.73, 84.49
Asefa et al. (2020)[32]	81.61	77.09, 86.13
Demelash et al. (2019)[38]	82.05	77.68, 86.41
Lencha (2018)[33]	81.50	76. 96, 86.04
Fedlu et al. (2019)[41]	81.46	76.91, 86.01
Lodebo et al. (2017)[36]	81.42	76.87, 85.98
Abdisa et al. (2021)[34]	81.10	76.53, 85.67
Aferu et al. (2020)[35]	82.69	78.68, 86.70
Tsegaye et al. (2016)[39]	81.12	76.55, 85.69
Tesfaye et al. (2019)[37]	81.59	77.07, 86.11
Ebuy et al. (2015[40]	81.18	76.60, 85.76
Tolossa et al. (2020)[19]	80.97	76.43, 85.51
Wondimu et al (2020)[20]	81.67	77.18, 86.17
Demissie et al. (2019)[21]	81.56	77.03, 86.09
Degavi et al. (2022)[23]	83.12	79.45, 86.78

### Subgroup analysis

Subgroup analysis was carried out based on the regions where the primary studies were performed. As a result, the highest prevalence was observed in Tigray with a prevalence of 91.12 (95% CI: 83.28, 98.96) and the lowest in Oromia with a prevalence of 79.31 (95% CI: 72.13, 86.49). Besides, the study population also checked subgroup analysis, and those studies that enrolled pregnant women had the highest prevalence, 82.26 (95% CI: 76.35, 88.17), while studies performed on both pregnant and lactating women had the lowest pooled prevalence of 80.80 (95% CI: 74.18, 87.42) (Table 3).

**Table 3:** Subgroup analysis of the level of adherence to option B+ program among HIV-positive women in Ethiopia.

Variables	Characteristics	Included studies	Number of study participants	Prevalence (95% CI)	I^2^(%), *P-value*
Region	Oromia	6	1,912	79.31 (72.13, 86.49)	99.1, <0.001
	SNNP	4	871	79.85(71.37, 88.33)	99.0, <0.001
	Amhara	2	460	81.47 (68.82, 94.11)	99.1, <0.001
	Tigray	2	318	91.12 (83.28, 98.96)	98.8, < 0.001
	Harari	1	190	83.20 (81.57, 84.83)	———
Study population	Pregnant	8	1, 903	82.26 (76.35, 88.17)	99.2, <0.001
	Pregnant and lactating	7	1,848	80.80 (74.18, 87.42)	99.2, <0.001
**Overall**		15	3,751	81.58 (77.33, 85.84)	99.1, <0.001

### Factors affecting the level of adherence to the option B+ program

The finding of the current systematic review and meta-analysis showed that the level of adherence to option B+ program among HIV-positive women was statistically associated with social and financial support, disclosure of HIV status to partner/family, time to reach health facility, counseling about drug side effects, experience of drug side effect, and knowledge of PMTCT.

**Fig 4:**
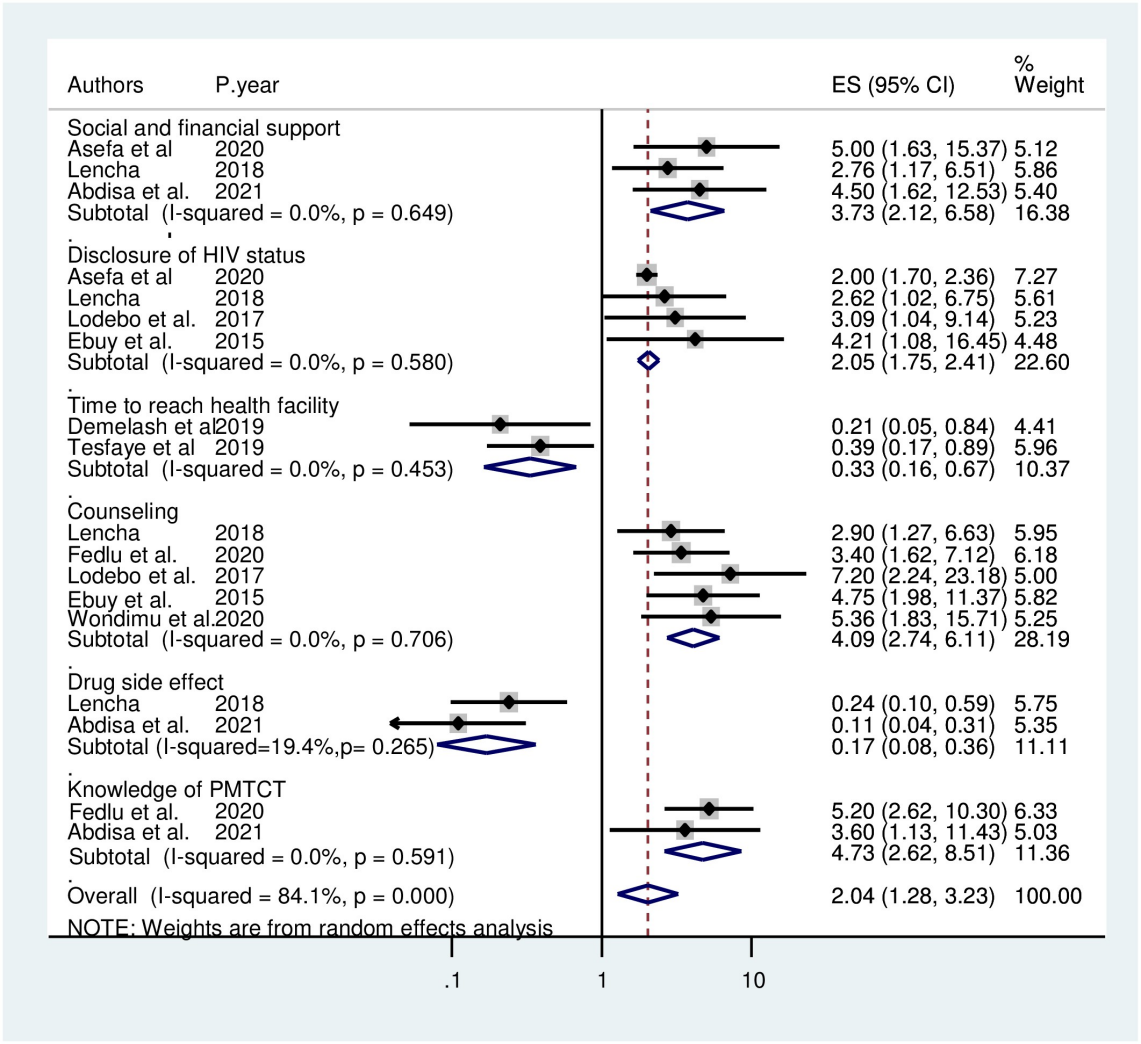
Factors affecting the level of adherence to the option B+ program among HIV-positive women in Ethiopia.

According to this meta-analysis study, three primary articles [32–34] showed that social and financial support was positively associated with adherence to the Option B+ program. The odds of adhering to the Option B+ program were 3.44 times (AOR = 3.73, 95% CI: 2.12, 6.58) higher among those who received social and financial support when compared to their counterparts. The heterogeneity test showed an I2 value of 0.0%, p = 0.41; hence, we used a random-effects model for analysis (Fig 4).

Four primary articles reported that disclosure of HIV status to their partner was significantly associated with adherence to the Option B+ program [32,33,36,40]. Women who disclosed their HIV status to their partner were two times (AOR = 2.05, 95% CI: 1.75, 2.41) more likely to adhere to the Option B+ program than those women who had not disclosed their HIV status to their partner. The heterogeneity test indicated that I^2^ = 0.0%, hence a random-effects model was applied for analysis (Fig 4).

Two primary articles [37,38] reported that time to reach health facilities was significantly associated with adherence to the Option B+ program. Women who should travel from 30 to 60 minutes on foot to access ART from service delivery facilities were 67% less likely to adhere to the option B + program (AOR = 0.33, 95% CI: 0.16, 0.67) than women who travel less than 30 minutes to access the facilities. The heterogeneity test indicated I^2^ = 0.0% and P = 0.29; hence, the random-effects model was applicable to yield the pooled odds ratio (Fig 4).

Based on the current findings, five studies [20,33,36,40,41] reported that counseling on ARV drug side effects and the health benefits of the drug was positively associated with good adherence. Compared with women who are not receiving proper counseling on ARV drug side effects, women who received proper counseling on the side effects and health benefits of drugs were four times (AOR = 4.09, 95% CI: 2.74, 6.11) more likely to adhere to the drug. Due to the absence of heterogeneity between the included studies (I^2^ = 0.0%, P = 0.70), a random-effects model was used for analysis (Fig 4).

As mentioned in two primary articles, adherence to the Option B+ program was also influenced by drug side effects. Those women who experienced side effects were 83% less likely to have adhered to the program (AOR = 0.17, 95% CI: 0.08, 0.36) when compared to those who did not experience any drug side effects. The heterogeneity test showed an I2 value of 15.5%, P = 0.28; hence, we used the random-effects model for analysis (Fig 4).

Lastly, two primary articles [34,41] revealed that having good knowledge regarding PMTCT was strongly associated with adherence to the Option B+ program. Women with good knowledge of PMTCT were nearly five times (AOR = 4.73, 95% CI: 2.62, 8.51) more likely to have adhered to the Option B+ program when compared to their counterparts. The investigators used a random-effects model for the analysis because the I^2^ value was 0.0%, p = 0.59 (Fig 4).

## Discussion

In many Sub-Saharan African countries, the Option B+ program has been accepted as a ‘paradigm-shifting innovation’ that has reshaped the way of HIV care and treatment for pregnant and lactating women [42]. However, its implementation continues to be challenged due to health system insufficiencies, which adversely influence women’s experiences and commitment to care [43,44]. The current systematic review was intended to estimate the pooled prevalence of adherence to the Option B+ program and factors affecting it among HIV-positive Ethiopian women. Accordingly, the pooled prevalence of an adequate level of adherence to the Option B+ program among HIV-positive Ethiopian women was 81.58% (95% CI: 77.33–85.84). This pooled prevalence report finding is consistent with other studies conducted in Zambia 82.5% [45], Malawi 80% [46], and Nigeria 79.3% [47]. However, the finding is lower than studies carried out in Kenya 89% [48], Malawi [49], and South Africa 89% [50]. The low level of option B+ program adherence reported in this meta-analysis might be due to discrepancies in socioeconomic status, the infrastructure of health care services, and a lack of promotion regarding PMTCT in the media. Adherence to the Option B+ program was also challenging due to high attrition rates, loss of follow-ups, stigma, and discrimination across the country [18,19].

However, the pooled level of adherence to the Option B+ program reported in this systematic review and meta-analysis is higher than the meta-analysis findings reported from low and middle-income countries 73.5% [51], Africa 69.3% [52], east Africa 71.8% [25], Uganda 76.8% [53], and Zimbabwe 67.7% [54]. The possible explanation might be due to time variation and greater emphases on improving maternal health in Ethiopia. Besides, the contextual differences in the study setting and population might also be responsible for the observed discrepancy. Once more, the comparable studies incorporated different socio-economic, cultural, and educational backgrounds in countries with different levels of adherence. The current finding implies that the level of adherence to the Option B+ program was inadequate when compared with the WHO recommendation, which suggested that an approaching 100% adherence is mandatory for optimal suppression of the viral load and MTCT [55]. Therefore, the involvement of multiple stakeholders, governmental and non-governmental organizations, is recommended to improve treatment compliance, reduce the risk of newborn infection, and improve the quality of life of mothers through viral load suppression. In addition, increasing the early initiation of antenatal care follow-up is helpful to pick up HIV-positive women and start treatment as soon as possible.

According to this systematic review and meta-analysis, women who have received social and financial support adhere well to the option B+ program for PMTCT service. This finding is consistent with reports illustrated by Indonesia [56], Africa [52], Tanzania [57], and Nigeria [58]. This might be due to the positive effect of social networking and financial support playing a greater role in promoting HIV-positive women’s psychological shift from resignation to a deeper acceptance of their status, which facilitated retention and adherence to their treatment. Besides, financial support might also include moral encouragement and health care assistance for transportation and equipping the home with different food remedies for balanced diets that impact healthy living in addition to the treatment [59].

Once more, social and financial assistance from friends, families, and the community helps the women to feel free and resist stigma and discrimination based on their status, as well as reminding them of the time for medication. Some societies believed that praying was the only means of healing rather than any medications mentioned as potential hindrances to consistent adherence to the treatment [60]. Hence, improving societal and financial support has a valuable effect on treatment adherence and minimizing new infections.

Women who disclosed their HIV status to partners, family, and friends were significantly associated with a good level of adherence to the option B+ program. The finding is comparable with studies conducted in Sub-Saharan Africa [61], Nigeria [47,58], Ukraine [62], Africa [52], east Africa [25], Uganda [53,60], and South Africa [63,64]. The possible explanation might be disclosing once sero status is an important tool to get social and psychological support to stick with their treatment. Disclosing HIV status is also an important parameter for controlling the spread of HIV infection, including the prevention of MTCT. Currently, both WHO [65] and the Center for Disease Control (CDC) [66] incorporate disclosing HIV status into their HIV counseling and testing protocols. In addition, disclosing one’s status also improves access to HIV prevention by motivating the partner for VCT and reducing risky sexual practices through the use of condoms to prevent transmission of HIV to their negative partner (discordant), which is also one additional benefit of option B+ over options A and B [67]. Moreover, disclosing HIV status helps to get reinforcement to continue taking the treatment. But if she didn’t disclose her status, she may hide her therapy due to fear of being observed by her partner and fear of taking her medication. Hence, there is a need to empower women to disclose their status to their partner, which has an advantage for early initiation of treatment and adherence to it.

In this meta-analysis, distance to reach health facilities is another strongly associated predictor of adherence. Women who traveled more than 30 minutes by foot to reach health facilities were 67% less likely to adhere to their treatment. The finding is consistent with study findings from the USA [68], Nepal [69], Malawi [46,49], and South Africa [64]. The possible explanation might be that costs like transport, food, and other accommodations may challenge women who travel more than 30 minutes to access health facilities. Once more, women from remote areas have difficulties transporting long distances, and the seasonal deterioration of poorer roads during the rainy season may hinder their ability to attain their schedule. Availing ART facilities in nearby areas and decentralizing HIV services by integrating with the main healthcare system are highly recommended for better retention.

Moreover, proper counseling regarding PMTC and the side effects of ART was also another significant predictor of good adherence to the Option B+ program. Findings from different studies also agree with the current report [25,51–53]. Counseling about the health benefits of option B+, like protection against mother-to-child transmission in future pregnancies and a continuing prevention benefit against sexual transmission to sero-discordant partners, encourages the women to stick with their treatment. Strict counseling on the importance of early initiation of option B+ has a positive impact on rapid initiation of the treatment as soon as diagnosed, which greatly impacts viral load suppression [70]. Besides, option B+ program also averted the load of child infection and adult sexual transmission of HIV and has great importance when compared with options A and B, so proper counseling on its special and unique significance helps the women adhere to the program [71]. Furthermore, appropriate counseling empowers the woman’s decision-making ability and builds confidence in their treatment, positively affecting adherence. Integrating and continuing PMTC counseling with other antenatal care services improves adherence to the treatment. However, nearly one-third of pregnant women 26% [72] have not attended ANC services, and as a result, they lack the benefit of counseling. Hence, increasing ANC service utilization is an essential component to improving treatment adherence since every woman has HIV testing and counseling services in their ANC care that ultimately scale up their level of adherence and improve their health and newborn outcome.

As mentioned elsewhere, adherence to the Option B+ program was negatively associated with drug side effects [49,52,53,62,73–75]. This finding is also evident in this meta-analysis. Those women who had experienced drug side effects were 83% less likely to have adhered to their treatment when compared with their counterparts. The possible rationale might be due to discomfort resulting from option B+ regimens (TDF-3TC-EFV), including headaches, strange dreams, and confusion, which mainly resulted from efavirenz, which may minimize their trust in the treatment and degrade their future hope, and finally, they may develop drug hesitation. Side effects such as lipodystrophy and weight loss are also challenging some women to stick with their treatment since these side effects change their body image. As a result, they feel that anyone may know that they are on ART [76]. Literature also noticed that patients who experienced lipodystrophy and weight loss discontinued their treatment to control their body changes [74]. Despite its minimal side effects, option B+ has numerous health benefits, including getting HIV-free newborns. Therefore, efforts on proper counseling regarding the benefit and side effects of the option B drug regimen are mandatory to scale up the uptake and adherence of it for PMTCT.

Lastly, this meta-analysis reported that knowledge of PMTCT was strongly associated with adherence to option B+. Women who had a good level of knowledge regarding option B+ program for PMTCT had a good level of adherence when compared to their counterparts. This finding is in line with a study conducted in Sub-Saharan Africa [61], Malawi [49], Ghana [77], and Nigeria [58]. This could be justified by the fact that having adequate and comprehensive knowledge regarding PMTCT may improve women’s awareness of the objectives and health benefits of taking and adhering to the treatment. Appropriate knowledge of PMTCT also prevents the woman from hesitating to utilize the option B+ treatment. Besides, women with some basic knowledge regarding PMTCT also understand the costs associated with not utilizing option B+.

Moreover, an adequate and acceptable level of knowledge about option B+ may increase women’s awareness, which can positively impact the overall healthy lives of women, newborns, and the large population. Therefore, improving women’s knowledge regarding the benefits of PMTCT is a paramount activity to scale up their level of adherence.

Even though this systematic review and meta-analysis tried to estimate the pooled national prevalence of adherence to the Option B+ program and identified some common predictors that are very important for program planners, it has limitations. First, some of the studies included in this systematic review and meta-analysis had small sample sizes, which may affect the actual level of option B+ adherence at the national level. Second, all Ethiopian regions were not represented in this systematic review and meta-analysis due to the limited number of studies in the country; only four regions were represented in this meta-analysis.

## Conclusion

This systematic review and meta-analysis showed that the level of adherence to the Option B+ program in Ethiopia is lower than the 95% level of adherence planned to be achieved in 2020. According to the results of this meta-analysis, social and financial support, disclosure of HIV status to a partner, time to reach the health facility, counseling on drug side effects, experience of drug side effects, and knowledge of PMTCT were significantly associated with the level of option B+ program adherence. The findings of this meta-analysis highlight that governmental, non-governmental, and other stakeholders need to design an effective strategy to scale up the level of disclosing one’s own HIV status, access health facilities, improve knowledge of PMTCT, and counsel the potential side effects of the option B+ drugs to enhance the adherence of the Option B+ program and advocate the program to reduce vertical transmission of HIV and multidimensional burdens related to HIV/AIDS diseases to women, families, and the community at large.

## Supporting information

S1 Table PRISMA checklist for systematic review and meta-analysis on level of adherence to the option B+ program and associated factors among HIV-positive women in Ethiopia. Systematic review and meta-analysis.
(DOC)

S2 Table Searching strategy for level of adherence to the option B+ program and associated factors among HIV-positive women in Ethiopia. (DOCX)

S3 Table Newcastle-Ottawa quality assessment scale for cross sectional studies to assess level of adherence to the option B+ program and associated factors among HIV positive women in Ethiopia. (DOCX)

## Abbreviations

AIDS: Acquired Immune Deficiency Syndrome
ANC: Antenatal Care
ART: Antiretroviral Therapy
HIV: Human Immune Virus
MTCT: Mother-to-Child Transmission
PMTCT: Prevention of Mother-to-Child Transmission
VCT: Voluntary Counseling and Testing
