# Supplementary material for: Level of adherence to option B+ program and associated factors among HIV-positive women in Ethiopia: A systematic review and meta-analysis
Source: PLoS One. 2024 Apr 25;19(4):e0298119. doi: 10.1371/journal.pone.0298119 (PMC11045077; doi:10.1371/journal.pone.0298119)
Supplement: S2 Table — (DOCX) [file pone.0298119.s002.docx]

**S2 Table:** Searching strategy for level of adherence to option B+ program and associated factors among HIV-positive women in Ethiopia.

| Databases | Searching terms | Number of studies |
| --- | --- | --- |
| PubMed | Level[All Fields] AND adherence[All Fields] AND option[All Fields] AND B[All Fields] AND program[All Fields] AND ("prevention and control"[Subheading] OR ("prevention"[All Fields] AND "control"[All Fields]) OR "prevention and control"[All Fields] OR "prevention"[All Fields]) AND ("infectious disease transmission, vertical"[MeSH Terms] OR ("infectious"[All Fields] AND "disease"[All Fields] AND "transmission"[All Fields] AND "vertical"[All Fields]) OR "vertical infectious disease transmission"[All Fields] OR ("mother"[All Fields] AND "child"[All Fields] AND "transmission"[All Fields]) OR "mother to child transmission"[All Fields]) AND ("hiv"[MeSH Terms] OR "hiv"[All Fields]) AND associated[All Fields] AND factors[All Fields] AND ("women"[MeSH Terms] OR "women"[All Fields]) AND ("Ethiopia"[MeSH Terms] OR "Ethiopia"[All Fields]) | 393 |
| Google scholar | Adherence" AND "option B" + AND "program" AND "prevention" AND "mother-to-child" AND "transmission" AND "HIV" AND "associated factors" OR "predictors" AND "HIV positive" AND "women" AND "Ethiopia" | 568 |
| HINARI | "Adherence" AND "option B " AND "program" AND "prevention of mother-to-child" AND "transmission" AND "associated factors" OR "determinants" OR "predictors” AND "HIV positive" AND "women" AND "Ethiopia" | 17 |
| Others databases |  | 10 |
| Total retrieved |  | 988 |
| Included |  | 15 |
