# Supplementary material for: Level of adherence to option B+ program and associated factors among HIV-positive women in Ethiopia: A systematic review and meta-analysis
Source: PLoS One. 2024 Apr 25;19(4):e0298119. doi: 10.1371/journal.pone.0298119 (PMC11045077; doi:10.1371/journal.pone.0298119)
Supplement: S3 Table — (DOCX) [file pone.0298119.s003.docx]

**S3 Table:** Newcastle-Ottawa Quality Assessment Scale for cross sectional studies to assess level of adherence to option B+ program and associated factors among HIV positive women in Ethiopia.

| Authors | Representativeness | Sample size | None-responders | Ascertainment | comparability | outcome | Quality score |
| --- | --- | --- | --- | --- | --- | --- | --- |
| Wondale et al. (2018) | 1 | 1 | 1 | 1 | 1 | 1 | 6 |
| Eyosiyas et al. (2020) | 1 | 1 | 1 | 2 | 1 | 1 | 7 |
| Yezina et al. (2019) | 2 | 1 | 1 | 2 | 1 | 1 | 8 |
| Mihratu (2018) | 1 | 1 | 1 | 2 | 1 | 1 | 8 |
| Anwar et al. (2019) | 2 | 1 | 1 | 1 | 2 | 1 | 8 |
| Juhar et al. (2017) | 2 | 1 | 1 | 1 | 2 | 1 | 8 |
| Samuel et al. (2021) | 1 | 1 | 1 | 2 | 1 | 1 | 7 |
| Temesgen et al. (2020) | 1 | 1 | 1 | 1 | 2 | 1 | 7 |
| Delelegn et al. (2016) | 2 | 1 | 1 | 1 | 2 | 1 | 8 |
| Dawit et al. (2019) | 1 | 1 | 1 | 1 | 2 | 1 | 7 |
| Haftamu et al.(2015 | 2 | 1 | 1 | 1 | 2 | 1 | 8 |
| Tadesse et al.(2020) | 1 | 2 | 1 | 1 | 2 | 1 | 8 |
| Fikadu et al (2020) | 2 | 2 | 1 | 1 | 1 | 1 | 8 |
| Dereje et al. (2019) | 2 | 2 | 1 | 1 | 1 | 1 | 8 |
| Girish et al. (2022) | 2 | 2 | 1 | 2 | 1 | 1 | 9 |

Interpretation of the score

Very Good Studies: 9-10 points

Good Studies: 7-8 points

Satisfactory Studies: 5-6 points

Unsatisfactory Studies: 0 to 4 points
